# Evaluation of Coseasonality of Influenza and Invasive Pneumococcal Disease: Results from Prospective Surveillance

**DOI:** 10.1371/journal.pmed.1001042

**Published:** 2011-06-07

**Authors:** Stefan P. Kuster, Ashleigh R. Tuite, Jeffrey C. Kwong, Allison McGeer, the Toronto Invasive Bacterial Diseases Network, David N. Fisman

**Affiliations:** 1 Department of Microbiology, Mount Sinai Hospital, Toronto, Ontario, Canada; 2 Dalla Lana School of Public Health, University of Toronto, Toronto, Ontario, Canada; 3 Department of Family and Community Medicine, University of Toronto, Toronto, Ontario, Canada; 4 Institute for Clinical Evaluative Sciences, Toronto, Ontario, Canada; 5 Department of Health Policy, Management and Evaluation, University of Toronto, Toronto, Ontario, Canada; Mahidol University, Thailand

## Abstract

**Background:**

The wintertime co-occurrence of peaks in influenza and invasive pneumococcal disease (IPD) is well documented, but how and whether wintertime peaks caused by these two pathogens are causally related is still uncertain. We aimed to investigate the relationship between influenza infection and IPD in Ontario, Canada, using several complementary methodological tools.

**Methods and Findings:**

We evaluated a total number of 38,501 positive influenza tests in Central Ontario and 6,191 episodes of IPD in the Toronto/Peel area, Ontario, Canada, between 1 January 1995 and 3 October 2009, reported through population-based surveillance. We assessed the relationship between the seasonal wave forms for influenza and IPD using fast Fourier transforms in order to examine the relationship between these two pathogens over yearly timescales. We also used three complementary statistical methods (time-series methods, negative binomial regression, and case-crossover methods) to evaluate the short-term effect of influenza dynamics on pneumococcal risk. Annual periodicity with wintertime peaks could be demonstrated for IPD, whereas periodicity for influenza was less regular. As for long-term effects, phase and amplitude terms of pneumococcal and influenza seasonal sine waves were not correlated and meta-analysis confirmed significant heterogeneity of influenza, but not pneumococcal phase terms. In contrast, influenza was shown to Granger-cause pneumococcal disease. A short-term association between IPD and influenza could be demonstrated for 1-week lags in both case-crossover (odds ratio [95% confidence interval] for one case of IPD per 100 influenza cases  = 1.10 [1.02–1.18]) and negative binomial regression analysis (incidence rate ratio [95% confidence interval] for one case of IPD per 100 influenza cases  = 1.09 [1.05–1.14]).

**Conclusions:**

Our data support the hypothesis that influenza influences bacterial disease incidence by enhancing short-term risk of invasion in colonized individuals. The absence of correlation between seasonal waveforms, on the other hand, suggests that bacterial disease transmission is affected to a lesser extent.

*Please see later in the article for the Editors' Summary*

## Abstract 2

Using a combination of modeling and statistical analyses, David Fisman and colleagues show that influenza likely influences the incidence of invasive pneumococcal disease by enhancing risk of invasion in colonized individuals.

## Editors' Summary

**Background:**

Although some pathogens (disease-causing organisms) cause illness all year round, others are responsible for seasonal peaks of illness. These peaks occur because of a complex interplay of factors such as the loss of immunity to the pathogen over time and seasonal changes in the pathogen's ability to infect new individuals. Thus, in temperate countries in the northern hemisphere, illness caused by influenza viruses (pathogens that infect the nose, throat, and airways) usually peaks between December and March, perhaps because weather conditions during these months favor the survival of influenza virus in the environment and thus increase its chances of being transferred among people. Another illness that peaks during the winter months in temperate regions is pneumonia, a severe lung infection that is often caused by *Streptococcus pneumoniae*. These bacteria can colonize the back of the throat without causing disease but occasionally spread into the lungs and other organs where they cause potentially fatal invasive pneumococcal disease (IPD).

**Why Was This Study Done?:**

Although the co-occurrence of seasonal peaks of influenza and IPD is well documented, it is unclear whether (or how) these peaks are causally related. For example, do the peaks of influenza and IPD both occur in the winter because influenza enhances person-to-person transmission of *S. pneumoniae* (hypothesis 1)? Alternatively, do the diseases co-occur because influenza infection increases the risk of IPD in individuals who are already colonized with *S. pneumoniae* (hypothesis 2)? Healthcare professionals need to know whether there is a causal relationship between influenza and IPD so that they can target vaccination for both diseases to those individuals most at risk of developing the potentially serious complications of these diseases. In this study, the researchers use several mathematical and statistical methods and data on influenza and IPD collected in Ontario, Canada to investigate the relationship between these seasonal illnesses.

**What Did the Researchers Do and Find?:**

Between January 1995 and October 2009, 38,501 positive influenza tests were recorded in Ontario by the Canadian national influenza surveillance network. Over the same time period, the Toronto Invasive Bacterial Diseases Network (a group of hospitals, laboratories, and doctors that undertakes population-based surveillance for serious bacterial infections in the Toronto and Peel Regions of Ontario) recorded 6,191 IPD episodes. The researchers used a mathematical method called fast Fourier transforms that compares the shape of wave forms to look for any relationship between infections with the two pathogens over yearly timescales (a test of hypothesis 1) and three statistical methods to evaluate the short-term effect of influenza dynamics on IPD risk (tests of hypothesis 2). Although they found wintertime peaks for infections with both pathogens, there was no correlation between the seasonal wave forms for influenza and IPD. That is, there was no relationship between the seasonal patterns of the two infections. By contrast, two of the statistical methods used to test hypothesis 2 revealed a short-term association between infections with influenza and with IPD. Moreover, the third statistical method (the Granger causality Wald test, a type of time-series analysis) provided evidence that data collected at intervals on influenza can be used to predict peaks in IPD infections.

**What Do These Findings Mean?:**

These findings support (but do not prove) the hypothesis that influenza influences IPD incidence by enhancing the short-term risk of bacterial invasion in individuals already colonized with *S. pneumoniae*, possibly by increasing the permeability of the lining of the airways to bacteria. By contrast, the lack of correlation between the seasonal wave forms for the two diseases suggests that person-to-person transfer of *S. pneumoniae* is affected by influenza infections to a lesser extent. These findings have important implications for disease control policy. First, they suggest that the increased number of influenza infections in pandemic years may not necessarily be accompanied by a marked surge in IPD. Second, because the findings suggest that some cases of IPD may be influenza-attributable, the extension of influenza vaccination to school-age children and young adults (a group of people at particular risk of IPD who are not normally vaccinated against influenza) could reduce the incidence of IPD as well as the incidence of influenza.

**Additional Information:**

Please access these Web sites via the online version of this summary at http://www.plosone.org/article/info:doi/10.1371/journal.pone.0015493

- A related research article by the same authors evaluating links between respiratory viruses and invasive meningococcal disease can be found in *PLoS One* (e0015493)
- The US Centers for Disease Control and Prevention provides information for patients and health professionals on all aspects of seasonal influenza and pneumococcal disease and pneumococcal vaccination
- The UK National Health Service Choices website also provides information for patients about seasonal influenza and pneumococcal infection
- MedlinePlus has links to further information about influenza and pneumococcal infections (in English and Spanish)
- FluWatch is the Canadian national surveillance system for influenza
- More information about the Toronto Invasive Bacterial Network is available
- The International Association for Ecology and Health provides information on the physical environment and its influence on health

## Introduction

The seasonal periodicity of pneumonia and influenza deaths in temperate countries is regarded as sufficiently commonplace that the term “flu season” is part of the English language vernacular. However, the widespread recognition of wintertime seasonality of severe respiratory disease obscures the fact that remarkably little is understood about the genesis of such seasonality [1],[2]. The wintertime co-occurrence of peaks in viral and bacterial respiratory disease, including severe invasive disease caused by *Streptococcus pneumoniae*, is well documented, but how and whether wintertime peaks caused by different pathogens are causally related is unknown. Existing work in this field has consisted largely of descriptive analyses that have evaluated week-by-week correlations between respiratory virus activity and invasive bacterial disease, with or without lags [3]–[5].

However, seasonal oscillation in disease incidence implies a complex system that includes such elements as loss of immunity and seasonally enhanced transmissibility with the latter potentially attributable to environmental, microbiological, or social forcing factors. Such a complex system cannot be adequately characterized by evaluating cross-sectional correlation in risk, particularly where causal links are concerned. Indeed, statistically significant correlation in risk when evaluating disease processes with shared seasonality is to be expected, and such correlation could be casual, as seen with many human respiratory pathogens with a peak incidence in winter, rather than causal.

We aimed to investigate the relationship between influenza infection and invasive pneumococcal disease (IPD) in Ontario, Canada, using several complementary methodological tools. We evaluated the relationship between the seasonal wave forms for influenza and IPD using fast Fourier transforms in order to examine the relationship between these epidemic waveforms over yearly timescales, i.e., to test the hypothesis that influenza influences IPD by enhancing person-to-person transmission. We also used three complementary statistical methods (time-series methods, negative binomial regression, and case-crossover methods) to evaluate the short-term effect of influenza dynamics on pneumococcal risk, i.e., to examine whether the risk of IPD is increased in previously colonized individuals.

## Materials and Methods

### Ethics Statement

The Toronto Invasive Bacterial Diseases Network (TIBDN) study was approved by Research Ethics Boards at all participating institutions. Ethical approval was not required for the remainder of this work.

### Population-Based Surveillance for Invasive Pneumococcal Infections

The Toronto Invasive Bacterial Diseases Network is a collaboration of all hospitals, microbiology laboratories, infection control practitioners, physicians, and public health units serving the population of metropolitan Toronto and Peel Regions (population 3.7 million), performing population-based surveillance for selected serious bacterial and viral infections [6]–[8]. All 25 hospitals, 19 laboratories, and 85 long-term care facilities serving residents of the population area participate in this network. All invasive pneumococcal infections identified in residents of Toronto/Peel from 1 January 1995 through 3 October 2009 were included in the analysis. Invasive pneumococcal infection was defined as illness in which *S. pneumoniae* was isolated from a normally sterile body site. When *S. pneumoniae* was isolated from specimens obtained from residents of the study area (defined by postal code), informed consent was obtained to collect isolates and detailed clinical data, including age, sex, and site of infection. Isolates were then sent to the central laboratory at Mount Sinai Hospital (Toronto), where they were confirmed as *S. pneumoniae* by standard methodology, including colonial morphology on blood agar, bile solubility, and susceptibility to optochin. Annual audits are conducted to ensure completeness of reporting.

### Surveillance for Influenza Infections

A national network of hospital and provincial laboratories submit weekly reports of numbers of tests performed (using viral culture or direct antigen detection) and numbers of positive tests for influenza A and influenza B to the Public Health Agency of Canada. For the purpose of this study, the surveillance data of the province of Ontario from 1 January 1995 through 3 October 2009 were included in the analysis.

### Environmental Exposure Data

We obtained time series data on ultraviolet (UV) radiation and weather from Environment Canada monitoring stations in the Greater Toronto Area [9],[10]. Where daily readings were taken at multiple locations, the arithmetic means of environmental data were used as exposure variables.

### Statistical Analysis

#### Evaluation of seasonal waveforms

The seasonality of disease occurrence was evaluated through calculation of autocorrelations for weekly case counts [2]. As yearly periodicity was observed with IPD, we estimated seasonal trends in disease occurrence of IPD and influenza A and B using Poisson and negative binomial regression models that incorporated sine and cosine oscillators, with 52-wk (annual) frequencies (i.e., incorporated fast Fourier transforms) [2],[11]. Using these parameters, the expression for the expected number of case counts for a given week, *E*(*Y*) is given by: *E*(*Y*)  = *e* ^α + β1•sin(2π(week/52))+β2•cos(2π(week/52))^. Accordingly, the phase-shift of the composite waveform generated by combining sine and cosine components can be approximated as tan^−1^(β_1_/β_2_) and can be used to estimate the timing of peak disease incidence [11]. Similarly, the amplitude (i.e., the maximum disease incidence) can be approximated as √(β_1_^2^ + β_2_^2^). Standard errors for phase-shift and amplitude were estimated as sums of standard errors for each model coefficient minus covariance. We evaluated the correlation between phase and amplitude terms for influenza A and B (combined), influenza A, and influenza B and pneumococcal sine waves, by year, by calculating Spearman correlation coefficients. Heterogeneity of phase and amplitude terms for *S. pneumoniae*, influenza A and B (combined), influenza A, and influenza B was assessed through meta-analysis using Cochran's Q test, and quantified using *I* ^2^ statistics. Differences in the magnitude of heterogeneity of amplitude or phase by pathogen was assessed using Knepp and Entwisle's method for assessing difference between chi-squared statistics [12]. As the 2003/2004 and 2008/2009 influenza seasons were outliers with respect to timing and amplitude, and we also performed sensitivity analyses in which these years were excluded.

#### Negative binomial regression models

As both deviance and Pearson goodness-of-fit statistics suggested over-dispersion of pneumococcal case counts, we evaluated the relationship between IPD rates, influenza, and environmental exposures using a series of negative binomial regression models [13]. Models incorporated population data derived from the 2001 and 2006 censes as offsets, with linear interpolation and extrapolation used to generate population denominators for noncensus years [14]. Model construction evaluating the impact of influenza A and B (combined), influenza A, or influenza B on IPD risk was performed by including viral infection incidence and environmental exposures considered a priori to be likely to influence pneumococcal disease epidemiology. Candidate exposures were based on prior observations by our group and previously published research by others [5],[15]–[17]. Environmental exposures included UV index, temperature, and relative humidity and both virological and environmental exposures were considered at lags of up to 4 wk prior to pneumococcal case occurrence, which we considered to be a biologically plausible window for effects. Models also incorporated seasonal and multi-year trend terms.

Once models had been fitted, we attempted to enhance model parsimony through model reduction, removing terms that had no influence on pneumococcal case counts, in a manner that minimized Akaike's information criterion (AIC), with final models representing those that best balanced parsimony and fit [18]. In models in which viral exposures were evaluated using either total influenza counts or influenza A counts, strong and statistically significant associations were observed between influenza burden, UV index, and relative humidity; we explored multiplicative interaction terms using categorical variables representing the highest quintile ranks of these exposures [19]. As noted above the 2004 and 2009 influenza seasons were outliers with respect to timing and amplitude, and we also performed sensitivity analyses in which models were reconstructed excluding these years.

#### Time series methods

Granger causality Wald testing was used to determine whether influenza A and B (combined), influenza A, and influenza B seasons can be used to forecast peaks in pneumococcal infections [20]. Originally, Granger methods were used in econometrics to evaluate the directionality of relationships between closely correlated time series; a causal exposure to an independent variable should be associated with a lagged change in the dependent variable, and this relationship should be absent when the temporal sequence of exposure and response is reversed. In other words, Granger “causality” can be used to postulate causality in linear prediction only if one thing happens before another, and not vice versa. It cannot be used to prove causation, but does provide important evidence that can support or refute causality.

#### Case-crossover analysis

We used a case-crossover approach to evaluate short-term associations between influenza A and B (combined), influenza A, and influenza B infection and IPD occurrence during influenza seasons in order to provide a means for evaluating the association between brief exposures and rare outcomes. The design is characterized by “self matching,” in that cases serve as their own controls. A “case” thereby is a day on which a case occurred, whereas a “control” is an adequately selected day on which a case did not occur [21],[22]. A time-stratified 2:1 matched case-crossover design in which hazard periods were defined as the reported date of IPD onset from the Toronto Invasive Bacterial Diseases Network database was incorporated. Beginning on January 1, 1995, the person-time at risk was divided into 3-wk time strata. Control periods were chosen by matching the hazard period by day of the week within the stratum, and could both precede, both follow, or straddle the hazard period [23],[24]. Random directionality of control selection was used in order to avoid biases occurring with unidirectional or uniform bidirectional control selection [24]. We evaluated the effects of influenza exposures and environmental covariates through construction of conditional logistic regression models with time lags from 0 to 4 wk.

Data were analyzed using Stata version 11.0 (Stata Corporation) and SAS version 9.1 (SAS Institute). *p*-Values of <0.05 were considered statistically significant.

## Results

**Figure 1:**
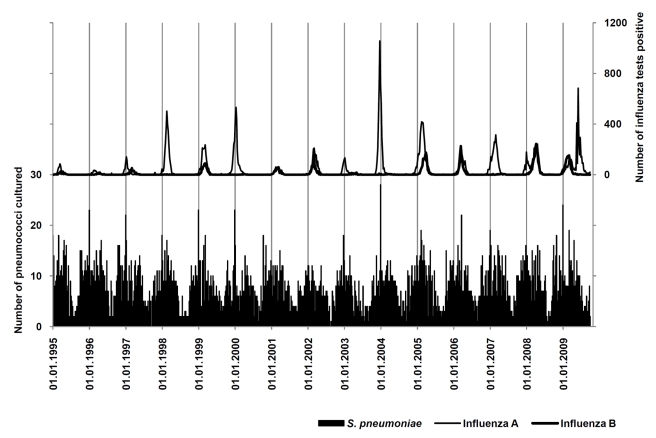
Surveillance for influenza A and B and IPD. Weekly numbers of IPD (black bars) and positive tests for influenza A (thin curve) and B (thick curve). in the areas under surveillance from January 1995 to October 2009.

The period from 1 January 1995 through 3 October 2009 was available for analysis, including a total number of 38,501 positive influenza tests in Ontario and 6,191 episodes of pneumococcal disease in Metropolitan Toronto/Peel Region (Figure 1).

### Periodicity of Influenza and Pneumococcal Disease

**Figure 2:**
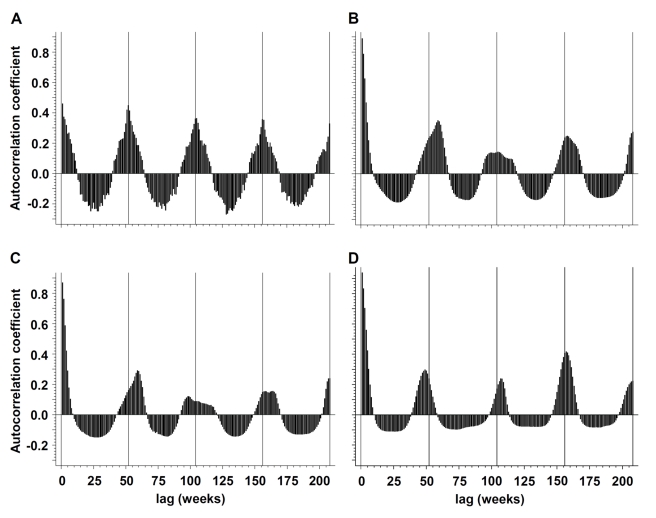
Periodicity of influenza and IPD. Periodicity of (A) IPD, (B) influenza A and B (combined), (C) influenza A, and (D) influenza B in the Toronto-Peel area, Ontario, Canada as illustrated by autocorrelograms, for the time period from January 1995 to October 2009.

Annual periodicity (i.e., recurrence at annual intervals) could be demonstrated for pneumococcal disease by spectral decomposition and construction of autocorrelations, with a maximum autocorrelation coefficient (ac)  = 0.4488 at week 52 (Figures 1 and 2). Peak incidence could be observed in week 6 (mid-February). Periodicity of influenza infections was less regular; the maximum ac could be identified at 59-week lags for influenza A and B (combined) (ac  = 0.3514), at 59-week lags for influenza A (ac  = 0.2922), and at 48-week lags for influenza B (ac  = 0.2903).

Negative binomial regression models revealed strong statistical evidence for annual oscillation for both pneumococcal disease and combined influenza A and B infections (*p* for nonlinear combination of sine and cosine oscillators for both pathogens <0.001). Whereas meta-analyses revealed substantial heterogeneity of phase (Cochran Q statistic: χ^2^ = 62810.15, df  = 15, *p*<0.001; *I* ^2^ = 100.0%) and amplitude (Cochran Q statistic: χ^2^ = 5752.86, df  = 15, *p*<0.001; *I* ^2^ = 99.7%) terms for influenza A and B (combined), there was less heterogeneity of amplitude (Cochran Q statistic: χ^2^ = 40.06, df  = 15, *p*<0.001; *I* ^2^ = 62.6%) terms for *S. pneumoniae*, and no significant heterogeneity for phase (Cochran Q statistic: χ^2^ = 23.28, df  = 15, *p* = 0.08; *I* ^2^ = 35.6%) terms. Differences in heterogeneity χ^2^ statistics between pathogens [12] were highly significant for both amplitude and phase terms (*p*<0.001). Heterogeneity of phase and amplitude terms of influenza A and influenza B was in line with influenza A and B (combined) (unpublished data). Findings were robust when outlier seasons (2003–2004 and 2008–2009) were excluded in sensitivity analyses.

Accordingly, no significant association between amplitude and phase of oscillatory waves of pneumococcal disease and influenza A and B (combined), influenza A, or influenza B could be detected using Spearman correlation (Tables 1–3).

**Table 1:** Spearman correlation coefficients for the association of phase and amplitude terms for influenza A and B (combined) and pneumococcal sine waves.

Phase or Amplitude	PhaseInfluenza A and B	Amplitude*S. pneumoniae*	Phase*S. pneumoniae*
Amplitude influenza A and B	−0.30 (0.26)	0.19 (0.49)	−0.35 (0.18)
Phase influenza A and B	—	−0.06 (0.83)	0.09 (0.75)
Amplitude *S. pneumoniae*	—	—	−0.49 (0.05)

Data are presented as Spearman correlation coefficient (*p*-value).

**Table 2 pmed-1001042-t002:** Spearman correlation coefficients for the association of phase and amplitude terms for influenza A and pneumococcal sine waves.

Phase or Amplitude	Phase Influenza A	Amplitude *S. pneumoniae*	Phase *S. pneumoniae*
Amplitude influenza A	−0.19 (0.48)	−0.06 (0.83)	−0.20 (0.05)
Phase influenza A	—	−0.11 (0.70)	0.12 (0.65)
Amplitude *S. pneumoniae*	—	—	−0.49 (0.05)

Data are presented as Spearman correlation coefficient (*p*-value).

**Table 3:** Spearman correlation coefficients for the association of phase and amplitude terms for influenza B and pneumococcal sine waves.

Phase or Amplitude	Phase Influenza B	Amplitude *S. pneumoniae*	Phase *S. pneumoniae*
Amplitude influenza B	−0.38 (0.15)	0.07 (0.80)	0.06 (0.84)
Phase influenza B	—	−0.29 (0.28)	0.06 (0.81)
Amplitude *S. pneumoniae*	—	—	−0.49 (0.05)

Data are presented as Spearman correlation coefficient (*p*-value).

### Acute Effects during Influenza Season

#### Negative binomial regression analysis

Results from negative binomial regression analysis suggest influenza A and B (combined) activity is associated with IPD (incidence rate ratio  = 1.09 case of IPD per 100 influenza cases, *p<*0.001), after adjustment for seasonal oscillation, multi-year trends, humidity, temperature, and UV index. Furthermore, there is an inverse association between average clear-sky UV index, both during the prior week, and 3 wk prior, and IPD (Table 4). Increased relative humidity was also associated with reduced risk of case occurrence. Models constructed using influenza A counts rather than total counts were identical in covariates and model performance to those constructed using total influenza counts (consistent with the large predominance of influenza A infections identified by the FluWatch system). We identified no significant interactions between influenza counts, UV index, and humidity. There was no significant association between influenza B incidence and IPD risk in either univariable or multivariable models. Model results were robust when we excluded influenza seasons that appeared to be outliers with respect to incidence (2003–2004 and 2008–2009) (unpublished data).

**Table 4:** Short-term effects of influenza dynamics on pneumococcal risk using multivariable negative binomial regression analysis.

Negative Binomial Regression Analysis	Incidence Rate Ratio	95% Confidence Interval	*p*-Value
Influenza A and B (combined, per 100 infections), 1-wk lag	1.092	1.047–1.138	<0.001
Influenza A and B (combined, per 100 infections), 3-wk lag	0.932	0.890–0.976	0.003
UVI average, 1-wk lag	0.927	0.893–0.963	<0.001
UVI average, 3-wk lag	0.946	0.917–0.976	<0.001
Relative humidity, 1-wk lag (%)	0.995	0.991–1.000	0.03
Mean temperature, 2-wk lag (°C)	0.993	0.984–1.002	0.14
Mean temperature, 4-wk lag (°C)	1.004	0.995–1.012	0.39

Model is also adjusted for year and seasonal oscillation.

UVI, clear-sky UV-index.

#### Granger causality Wald test

Based on the Granger causality Wald test, there is evidence that influenza A and B (combined) Granger-cause pneumococcal disease (Chi-square  = 23.28, df  = 2, *p<*0.001). These results could be reproduced for influenza A (*p<*0.001) and influenza B (*p* = 0.017). Conversely, pneumococcal disease could not be shown to Granger-cause influenza A and B (combined) infection (Chi-square  = 4.39, df  = 2, *p* = 0.11).

#### Case-crossover results

We were able to identify a significant association between total influenza (A and B) and IPD with a 1-wk lag using a case-crossover approach (odds ratio [95% confidence interval] for one case of IPD per 100 influenza cases, 1.10 [1.02–1.18]) (Table 5). There was no significant association between IPD risk and daily changes in clear-sky UV index, temperature, or relative humidity.

**Table 5:** Short-term associations between influenza A and B and IPD according to case-crossover methods.

Lag (wk)	Odds Ratio	95% Confidence Interval	*p*-Value
0	1.05	0.97–1.13	0.20
−1	1.10	1.02–1.18	0.01
−2	1.00	0.93–1.09	0.90
−3	0.93	0.86–1.01	0.07
−4	1.02	0.94–1.11	0.65

Data are presented as odds ratios with 95% confidence intervals for 1 IPD case per 100 influenza cases.

## Discussion

Although this observational study cannot prove epidemiological causality, our analysis of seasonal oscillation in IPD and influenza A and B incidence in Ontario, Canada, is strongly suggestive of a causal relationship between influenza and IPD. The fact that we did not find a relationship between seasonal dynamics of influenza and IPD suggests that this relationship likely results from effects of influenza on risk of invasive disease in individuals with pneumococcal colonization. We found no epidemiological evidence to support the concept that influenza has a strong effect on IPD risk via changes in pneumococcal transmission dynamics that would be caused by enhanced susceptibility to pneumococcal colonization, increased infectiousness of pneumococcus-colonized individuals, or increased duration of carriage. All such changes would be expected to perturb the dynamics of pneumococcal seasonality in such a way that pneumococcal seasonal waves would “track” influenza waves. While we found significant and temporally directional relationships between influenza burden in the population and IPD risk, the seasonal “waves” of influenza and IPD were independent of one another, with substantial variability in influenza contrasted with the far more regular seasonal occurrence of IPD. The nonstereotyped nature of wintertime seasonality of viral respiratory pathogens has been noted previously [17].

While prior work has evaluated the impact of influenza infection on pneumococcal risk, much of this work has been performed in experimental rodent models that may not be generalizable to human populations [25]–[27]. A number of epidemiological analyses have been performed; many have failed to account for either confounding by other seasonal factors, or “ecological fallacy” resulting from the use of aggregated exposure and outcome data [3]–[5],[28]. More methodologically sophisticated studies have yielded conflicting results on the link between influenza and IPD [29]–[31].

The major clinical implications of our study are 2-fold: first, that dramatic increases in influenza incidence, as might be seen during a pandemic year, would not in and of themselves imply a marked surge in risk of IPD, but rather would do so only if there were overlap between flu and pneumococcal waves. This observation may help explain the absence of a marked increase in IPD risk in our jurisdiction during the (atypical) spring wave of the 2009 influenza A-H1N1 pandemic. Although public health agencies issued calls for stepped-up pneumococcal vaccination because of the pandemic [32], the fact that the pandemic and pneumococcal season were out of phase may have made this unnecessary. Second, the short-term surge in IPD risk accompanying lagged increases in influenza, if causal, implies that some fraction of IPD is “influenza-attributable.” As such there may be important unrecognized population health benefits associated with influenza vaccination programs that include younger individuals. Traditionally, influenza immunization programs have focused on older individuals and sometimes the very young, as the risk of adverse outcomes of flu appear greatest in these age groups. Some modelers have recommended younger individuals be targeted with influenza vaccination in order to protect older individuals from influenza via herd effects, and these projections appear to have been borne out in at least one recent randomized trial [33],[34]. However, IPD causes important morbidity in those in age groups not included in traditional influenza immunization programs, and our analysis suggests that prevention of influenza-attributable pneumococcal disease could also be considered as a benefit of influenza vaccination targeting older children and younger adults.

If our findings are correct, and influenza increases the risk of IPD without influencing pneumococcal transmission dynamics, the question remains: what are the drivers of the remarkably stereotyped seasonality of IPD? Crude correlations between IPD risk and mean weekly temperature and mean weekly minutes of darkness, but not precipitation, have been reported by Dowell et al. [35]. A previous study by our group of environmental determinants of IPD risk in Philadelphia [22], identified diminished ambient UV radiation as a significant predictor of increased IPD risk, an effect that could either be due to effects of UV radiation on 1,25-(OH)_2_-vitamin-D metabolism, or direct mutagenic effects on vegetative bacteria. The current study, performed in a different jurisdiction, replicates this finding.

Like any observational study, ours has several limitations. As it is not possible to randomize exposure to influenza, or to meteorological exposures, observational studies like this one are necessary to evaluate the impact of such exposures on invasive bacterial disease risk in real-world human populations. Limitations of observational designs like the one we have used here include difficulties with residual confounding, ecological fallacy due to aggregation of exposures, and measurement issues. While it is not possible to control for all residual confounding by unmeasured confounders in regression models, our use of seasonal smoothers, multivariable techniques, and case-crossover design should largely have controlled for confounding of influenza effects by seasonally varying factors including seasonal behaviors and meteorological effects. Our use of case-crossover design implicitly matches for all seasonal effects that would be constant over the 3-wk time block used for each risk stratum. The case-crossover approach assumes that the distribution of exposure is constant across referent times, which should be controlled by careful referent selection. It can be associated with bias if referent periods are not chosen a priori or if referents are functions of the observed event times [36]. In order to circumvent this so-called “overlap bias,” control periods in our analysis were chosen by matching the hazard period by day of the week within the 3-wk stratum, and could both precede, both follow, or straddle the hazard period [24].

Although implied by its name, Granger causality testing does not prove true causation in the epidemiologic sense. It originally is an econometric method to infer predictive value of one time series over another and thus, rather than prove causation, adds a valuable piece to the overall interpretation of our analyses towards potential causation. With respect to ecological effects, it should be noted that we effectively treat influenza as an aggregate “environmental” exposure, in that we don't have influenza status on the individuals who develop invasive bacterial disease, which creates the risk of ecological effects, including ecological fallacy, in our study. There are practical barriers to formulating an adequately powered study that actually captured data on the temporal relationship between influenza and invasive bacterial disease at the level of the individual (e.g., through ongoing evaluation of individuals in the population for influenza infection, with subsequent identification of the [rare] individuals who developed invasive bacterial disease).

Finally, with respect to measurement issues, for influenza A and B, we had to rely on public health surveillance data that are incomplete both because of under-reporting, and also because a majority of individuals with influenza never undergo virological testing [37]. Other exposures may have been misclassified because of measurement or population mobility. This misclassification should not have introduced bias into our study in the absence of correlation between pneumococcal risk and influenza or meteorological reporting but may have reduced our statistical efficiency. Nondifferential misclassification of exposures would mean that the effects reported here are likely to be lower-bound effects, with true effects being larger. Furthermore, the strong degree of agreement in our findings, across multiple methodological approaches, suggests that they are robust and not artifacts of the analytic approach taken.

In conclusion, our data support the hypothesis that influenza influences IPD risk by enhancing pneumococcal invasion in colonized individuals, but has little effect on the transmission dynamics of pneumococcal infection. We suggest that the mechanism for such an effect might be influenza-related alterations at the level of the respiratory epithelium [27]. These effects appear distinct from those of ambient environmental effects, although the effect of increased UV radiation in reducing IPD risk that we had previously reported in a study performed in Philadelphia needs further study [22]. These findings have important implications for disease control policy, and suggest that improved efficacy of influenza vaccines [38], and novel vaccination strategies that more effectively control influenza by vaccinating younger individuals [33], could have important effects in reducing IPD as well.

## Abbreviations

ac: autocorrelation coefficient
IPD: invasive pneumococcal disease
UV: ultraviolet
